# 

**Published:** 1851-08

**Authors:** 


☞ Our readers will observe that an *Eclectic Department* does not appear in this No. This is owing to want of room! We hardly know whether the omission requires an apology, or not. Notwithstanding we relinquish nearly all our space to original contributions, we have received several papers which are necessarily deferred. Our next No. will contain articles by Prof. Hamilton, Dr. House, and Dr. Colegrove, which are already on file. We shall also commence in our next No. the publication of an essay on the Medical Profession, by Dr. Wood, U. S. Navy, with which we have been favored by the author.

---

*A Fair Hit.* — An exchange says: " It has been said, that in spite of all the medical science and systems of the day, a sick minister, who has a rich congregation, can only be cured by a voyage to Europe. A singular fact in therapeutics."—*Med. Gazette.*

